# Functional Foods with Modulating Action on Metabolic Risk Factors

**DOI:** 10.3390/foods12214043

**Published:** 2023-11-06

**Authors:** José Luiz de Brito Alves, Evandro Leite de Souza

**Affiliations:** Department of Nutrition, Federal University of Paraiba, João Pessoa 58051-900, PB, Brazil; jose.luiz@academico.ufpb.br

Health-related metabolic risk factors, such as elevated blood pressure, hyperglycemia, obesity, and dyslipidemia, can lead to metabolic syndrome and increased risk of cardiovascular disease, stroke, and death. In recent years, there has been growing evidence that functional foods, food components, and bioactive molecules of plant, animal, and microbial origins can exert preventive and therapeutic benefits for human health by modulating host metabolism, physiology, nutrition, and immune functions. Therefore, this Special Issue addressed several aspects of functional foods with modulating action on metabolic risk factors.

Jamrozik et al. [1] carried out a review on *Hibiscus sabdariffa* in diabetes prevention and treatment. Using studies carried out with animals and clinical trials, the authors showed that *H. sabdariffa* exerts a great hypoglycemic property and induces beneficial effects on the pancreas, kidney, and liver, in addition to having lipidic and blood pressure-lowering effects. A systematic review conducted by Arisanti et al. [2] showed that sweet potato (*Ipomoea batatas*) is effective in the treatment of type 2 diabetes and suggested that sweet potato may increase insulin secretion and insulin sensitivity, suppress hepatic glucose production, and inhibit inflammatory pathways.

A systematic review of clinical trials by Silva et al. [3] evaluated the effects of regular consumption of Brazil nuts (*Bertholletia excelsa* H.B.K.) on health markers. The scientific evidence of this systematic review showed that Brazil nut consumption exerts beneficial effects on antioxidant status, lipid profile, and inflammatory biomarkers in healthy subjects, as well as decreases oxidative stress and inflammatory biomarkers in patients with obesity, type 2 diabetes, and coronary artery disease undergoing hemodialysis and with cognitive impairment. 

Lima et al. [4], through a review paper, showed that Brazilian native fruits, such as acerola, açaí, baru, buriti, guava, jabuticaba, juçara, and passion fruit, and their by-products are rich in bioactive compounds, including soluble and insoluble fiber and a variety of phenolic compounds. The authors gathered information showing that Brazilian native fruits and their by-products can exert beneficial effects on gut microbiota composition with promising repercussions in treating and preventing non-communicable chronic diseases.

Functional foods are considered potential adjuvants in therapeutic options for cardiometabolic disease in pregnancy [5,6]. A review carried out by Trindade-da-Costa et al. [7] reported the effects of dietary supplementation of quercetin in the treatment of signs and symptoms of cardiometabolic diseases during pregnancy, including gestational diabetes mellitus, dyslipidemia, hypertensive syndrome, and maternal overweight. The authors suggested that although trials have not been carried out in pregnant humans, dietary consumption of quercetin-rich fruits and vegetables, including dill, apple, berries, grapes, broccoli, onion, spinach, and oregano, could be an effective planned dietary regimen to decrease the risks associated with cardiometabolic diseases during pregnancy.

In addition to critical reviews, this Special Issue published new findings on functional foods with modulating effects on metabolic risk factors. Xu et al. [8] showed that administration of hydroxyl-α-sanshool, a long-chain unsaturated fatty acid amide extracted from *Zanthoxylumbungeanum* Maxim fruit (0.8 mg/kg for 28 days), improved the richness and diversity of gut microbiota, decreased the *Firmicutes*/*Bacteroidetes* ratio, and reduced glycosylated hemoglobin and diabetic dyslipidemia in streptozotocin-induced insulin resistance in rats. Lucena et al. [9], investigating the functional properties of yellow mombin (*Spondias mombin*), showed that yellow mombin supplementation (400 mg/kg for two weeks) decreased dyslipidemia and oxidative stress in rats fed a high-fat diet. Cheng et al. [10] developed an herbal formula consisting of three ingredients (bainiku-ekisu, black garlic, and *Mesona procumbens* Hemsl), named Mei-Gin formula. The authors administered Mei-Gin Formula to high-fat diet-induced obese rats at three doses (50, 100, and 300 mg/kg rat) for eight weeks. The results showed that Mei-Gin formula supplementation exerted anti-obesity effects by increasing lipolysis, fatty oxidation, and thermogenesis pathways. The development of functional foods combining probiotics with polyphenols for host health benefits is a growing research area [11,12]. Harahap et al. [13] used a product combining the probiotic *Lactobacillus acidophilus* DSM20079 with the isoflavones daidzein and genistein. The authors reported that daily consumption of a soy-based diet rich in isoflavones and probiotics for eight weeks increased magnesium status in healthy female rats. Finally, Kumar et al. [14], using a predictive model, showed that *Lactiplantibacillus plantarum* MTCC25432 is a strain with great riboflavin-producing potential.

In summary, this Special Issue provides evidence that several functional foods could exert significant modulating effects in preventing and alleviating the symptoms of several metabolic disorders, mainly by reducing oxidative stress and affecting inflammatory pathways.
